# Investigation of the Prognostic Significance of Vasculogenic Mimicry and Its Inhibition by Sorafenib in Canine Mammary Gland Tumors

**DOI:** 10.3389/fonc.2019.01445

**Published:** 2019-12-19

**Authors:** Maria Carolina Mangini Prado, Sofia de Almeida Losant Macedo, Giulia Gumiero Guiraldelli, Patricia de Faria Lainetti, Antonio Fernando Leis-Filho, Priscila Emiko Kobayashi, Renee Laufer-Amorim, Carlos Eduardo Fonseca-Alves

**Affiliations:** ^1^Department of Veterinary Surgery and Anesthesiology, São Paulo State University—UNESP, Botucatu, Brazil; ^2^Department of Veterinary Clinic, São Paulo State University—UNESP, Botucatu, Brazil; ^3^Institute of Health Sciences, Universidade Paulista—UNIP, Bauru, Brazil

**Keywords:** angiogenesis, dog, breast cancer, tubular assay, antiangiogenic drugs

## Abstract

Canine mammary gland tumor (CMT) is one of the most important tumors in intact female dogs, and due its similarity to human breast cancer (BC), it is considered a model in comparative oncology. A subset of mammary gland tumors can show aggressive behavior, and a recurrent histological finding is the presence of vasculogenic mimicry (VM). VM is a process in which highly aggressive cancer cells fuse, forming fluid-conducting channels without endothelial cells. Although, VM has been described in canine inflammatory carcinoma, no previous studies have investigated the prognostic and predictive significance of VM in CMT. Thus, this research aimed to investigate the prognostic significance of VM *in vivo* and the capacity of sorafenib to inhibit VM *in vitro*. VM was identified *in situ* in formalin-fixed paraffin-embedded CMT samples (*n* = 248) using CD31/PAS double staining. VM was identified in 33% of tumors (82/248). The presence of VM was more strongly related to tumor grade than to histological subtype. Patients with positive VM experienced shorter survival times than dogs without VM (*P* < 0.0001). Due to the importance of the VEGF-A/VEGFR-2 autocrine feed-forward loop in epithelial tumors, we investigated the association between VEGF-A and VEGFR-2 expression by neoplastic tumor cells and the associations of VEGF-A or VEGFR-2 expression with VM. Among the VM-positive samples, all (*n* = 82) showed high scores (3 or 4) for VEGF-A and VEGFR-2, indicating that VM was a common finding in tumors overexpressing VEGF-A and VEGFR-2. Thus, we cultured two CMT primary cell lines with VM abilities (CM9 and CM60) *in vitro* and evaluated the anti-tumoral effect of sorafenib. The CM9 cell line showed a half maximal inhibitory concentration (IC_50_) of 2.61 μM, and the CM60 cell line showed an IC_50_ of 1.34 μM. We performed a VM assay *in vitro* and treated each cell line with an IC_50_ dose of sorafenib, which was able to inhibit VM *in vitro*. Overall, our results indicated that VM was a prognostic factor for dogs bearing CMT and that sorafenib had an inhibitory effect on VM in CMT cancer cells *in vitro*.

## Introduction

Canine mammary gland tumor (CMT) is one of the most common tumors in intact female dogs and is a therapeutic challenge due to its metastatic rate (1). In a One Health perspective, CMT was considered a spontaneous model for studying human breast cancer (BC) (2). Thus, studies on CMT can benefit both humans and dogs. CMT and human BC share many clinical and pathological similarities, including hormonal regulation (2). To grow and metastasize, tumor cells require a sufficient supply of nutrients and oxygen (3). The process of forming new vessels from existing ones, known as neoangiogenesis, is induced by hypoxia and the production of proangiogenic factors (4). Among angiogenic factors, vascular endothelial growth factor-A (VEGF-A) overexpression and its receptor (VEGFR-2) play key roles (3, 4). VEGF-A/VEGFR-2 deregulation was previously demonstrated in canine CMT (2) and human BC (5).

Vasculogenic mimicry (VM) is defined as a process used by highly aggressive neoplastic cells to generate vascular-like structures without the presence of endothelial cells (6). VM has been extensively described in various tumors and participates in tumor spread and metastasis (6–9). Many signaling mechanisms are involved in the initiation of VM. Molecules that are involved in this process are being investigated with the aim of developing new strategies for therapeutic targets against cancer (6). Although, the mechanism of VM is not yet clear, studies have found that the ERK-1/PI3K/MMP-2 signaling pathway may be critical. In addition, VEGFR-2 can induce proliferation through activation of the canonical extracellular signal-regulated kinase (ERK) pathway. Therefore, VEGFR-2 expression by tumor cells may be associated with VM formation (10). Common anti-angiogenic drugs primarily target endothelial cells by inducing apoptosis in these cells and reducing the proliferation of aggressive tumors (6). Among antiangiogenic therapies, sorafenib is a tyrosine kinase inhibitor widely used in human medicine (11–13) that was recently used in veterinary medicine (14).

In humans with liver cancer, sorafenib has been shown to effectively inhibit angiogenesis and induce apoptosis, with good antitumor effects (15). Lee et al. (16) used sorafenib to inhibit the development of human BC cell lines and showed effective induction of apoptosis and autophagy, indicating the potential of sorafenib in human patients with BC. However, to our knowledge, there are no previous studies investigating the antitumor effect of sorafenib on canine mammary cancer cell lines. Several researchers have investigated models to study MV *in vitro* (3, 9, 17). Due to the importance of VM in the development of cancer metastasis and the relation of VM with patient prognosis, this research aimed to verify the role of VM in canine mammary tumors *in vitro* and evaluate the association between VEGF-A/VEGFR-2 expression in canine mammary carcinoma tumor samples. In addition, we evaluated the inhibitory effect of sorafenib on VM in canine mammary gland tumor cells *in vitro*.

## Methods

### Study Design

**Figure 1:**
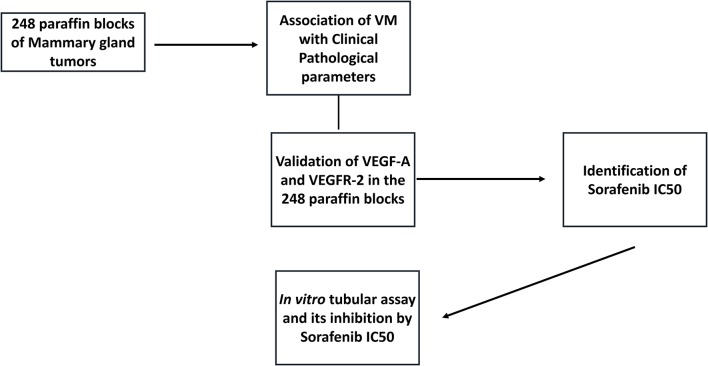
Graphic representation of the study design. We selected 248 mammary gland tumors from the veterinary pathology service, and samples were evaluated for the presence of vasculogenic mimicry (VM). Then, we performed CD31/PAS double staining to identify VM structures, confirming that samples were positive for VM. Two canine mammary gland tumor cell lines were selected, and an *in vitro* tubular assay was performed to identify the cellular VM ability. Based on the VEGF-A and VEGFR-2 immunohistochemical analysis, we selected VEGF-A/VEGFR-2 and validated their expression in our tumor group. After confirming the associations of VEGF-A/VEGFR-2 expression with VM, we performed *in vitro* assays to evaluate the ability of sorafenib (a VEGFR-2 inhibitor) to inhibit VM *in vitro*.

This study was performed in accordance with national and international guidelines for the use of animals in research. All procedures were approved by the institutional Ethics Committee for the Use of Animals (protocol number: CEUA 0091/2018). The experiment was designed in two steps. First, we selected cases of canine mammary gland tumor from the archives of the Veterinary Teaching Hospital of São Paulo State University (UNESP) between 2008 and June 2019. These cases were used to evaluate the associations of vasculogenic mimicry with clinical pathological information. The study design is detailed in Figure 1.

### Patients

We retrospectively included 248 canine mammary gland tumor-bearing dogs treated with surgery, with or without chemotherapy. Our inclusion criteria were treatment with surgery with or without chemotherapy, clinical information available in patient records, the presence of a paraffin block in the veterinary archive for immunohistochemical evaluation and no chemotherapeutic treatment prior to surgery. Histological classification was performed according to Goldschimidt et al. (1), and tumor histological grading was performed according to Karayannopoulou et al. (18). For clinical evaluation, the patients underwent a complete blood count, abdominal ultrasound and three-view thoracic radiographic examination. The clinical stage of disease was established according to the World Health Organization classification for CMT (stages I–IV), as modified by Sorenmo et al. (19). Patients with at least stage III and tumor histological grade II disease received adjuvant treatment, and patients with metastatic disease at diagnosis were treated with chemotherapy. Clinical follow-up was performed according to Dos Anjos et al. (2).

### CD31-Periodic Acid Schiff (PAS) Double Staining for VM

All procedures for CD31/PAS double staining were performed according to the protocol by (20). Briefly, tissue sections (*N* = 248) were stained using a rabbit polyclonal anti-CD31 primary antibody (PECAM-1, Thermo Fischer Scientific, Waltham, MA, EUA) for blood endothelial cell identification using a polymer system conjugated with peroxidase as the first staining step. Then, the sections were counterstained with 0.5% PAS and Schiff. The criteria for determining CD31- and/or PAS-positive VM and procedures for positive/negative control were those described by (20). VM was characterized by the formation of tubular or fracture-like structures by tumor cells containing red blood cells with positive CD31 and/or PAS expression (20).

### VEGF-A and VEGFR-2 Immunohistochemistry

Because we found *VEGF* deregulation by and previous publication (2), we performed immunohistochemistry to detect VEGF-A and VEGFR-2 expression in the 248 tumor samples used to evaluate VM formation and prognosis. The procedures for VEGFR-2 immunohistochemical detection and evaluation and controls were previously described by our research group (2). VEGF-A immunostaining was performed using a mouse monoclonal antibody (clone VG1, Dako Cytomation, Carpinteria, CA, USA). Antigen retrieval was achieved by incubation in a citrate buffer pH 6.0 in a pressure cooker (Pascal, Dako, Carpinteria, CA, USA), and endogenous peroxidase activity was blocked with 8% hydrogen peroxide diluted in methanol for 10 min. Then, the samples were incubated with the primary antibody overnight, followed by incubation with a polymer system (Envision, Dako, Carpinteria, CA, USA) for 1 h. The samples were incubated with 3,30-diaminobenzidine (DAB; Dako, Carpinteria, CA, USA) for 5 min and counterstained with Harris haematoxylin for 1 min. The blood vessels in the tumor samples were used as an internal positive control. For the negative control, mouse (Negative Control Mouse, Dako, Carpinteria, CA, USA) immunoglobulin was used to stain a new CMT section. All antibodies were cross-reacted with canine tissue provided by the manufacturer. For the immunohistochemical analysis, the evaluators (MCMP and CEFA) were blinded to patient clinical data, histological type and grade.

### Primary Cell Culture and the Anti-tumoural Effect of Sorafenib

The establishment of canine mammary cell cultures followed the previous description published by our research group (21), and all procedures for the establishment, characterization and culture of CM9 and CM60 mammary primary cells were described previously (22). The anti-tumoural effect of sorafenib was determined by an assay based on the cleavage of an MTT salt into purple crystals by metabolically active cells. For this experiment, each cell line was seeded in a 96-well plate specific for cell culture containing DMEM F12 (Lonza, Basel, Switzerland) supplemented with 10% FBS (Lonza, Basel, Switzerland) and 1% penicillin and streptomycin. The cells were maintained for 24 h at 37°C. After this initial period, the cells were cultured and incubated in medium without serum, and sorafenib was added to the medium at 2, 4, 6, 8, 10, 12, 14, or 16 μM for 24 h. For MTT controls, we used untreated cells (basal control) and cells treated with the highest DMSO concentration (control for DMSO toxicity). In the same plate, each dose was tested in triplicate, and each replicate was performed in triplicate (3×3). After a 24-h incubation, 10 μL of MTT labeling reagent was added to each well, and the plate was incubated at 37°C for 4 h. Then, the cultures were solubilized, and the spectrophotometric absorbance of the samples was detected using a microtiter plate reader at 570 nm.

### *In vitro* VM Assay and the Sorafenib Antitumor Effect

This experiment was based on two steps. First, we evaluated cell cultures to determine the time point with the most VM formation by the two CMT cell lines (CM9 and CM60). After determining this time point, we treated the cells with sorafenib (2.61 μM for CM9 cells and 1.34 μM for CM60 cells). All experiments were performed in triplicate with negative controls (cells treated with DMSO).

All experiments were performed with 80% confluent cell cultures. Three-dimensional (3D) cell cultures were prepared in a 24-well plate. In total, 200 μl of Matrigel (Matrigel^®^ Growth Factor Reduced (GFR) Basement Membrane Matrix, ^*^LDEV-Free, Corning, New York, NY, USA) was added to each well and air-dried for 30 min at room temperature. Then, the cell cultures were trypsinized, and 50,000 cells were suspended in 500 μl of DMEM without fetal bovine serum and seeded in each well. The cells were incubated in a humidified atmosphere with 5% CO_2_ at 37°C. The cells were evaluated for VM with an inverted microscope at 1, 2 3, 4, 5, 6, and 7 h.

The cells showed the best tubular structure formation at 4 h. Thus, we performed 3D experiments in triplicate as described above. However, we seeded cells in a 24-well plate with sorafenib at the IC50 dose for each cell line. After 4 h, we evaluated VM formation with an inverted microscope, comparing treated cells with control cells. The control cells were seeded in triplicate and treated by adding the DMSO concentration of the IC50 dose for each cell line.

### Statistical Analysis

Clinicopathological data were evaluated in a descriptive way, with the data presented as percentages. We evaluated patient survival in the context of clinicopathological data, including the presence of VM and the expression of VEGF-A and VEGFR-2. Survival curves were generated using Kaplan-Meier analysis. Chi-square or Fisher exact tests were used to evaluate the correlations of VEGF-A and VEGFR-2 expression with clinicopathological parameters. Samples with scores of 1 or 2 were considered to have low VEGF-A or VEGFR-2 expression, and samples with scores of 3 or 4 were considered to have high expression. Statistical analysis was performed using GraphPad Prism v.8.1.0 (GraphPad Software Inc., La Jolla, CA, USA).

## Results

### Clinical Information

Two hundred twenty-three patients (223/248) had malignant mammary gland tumors, and the remaining 25 patients had benign tumors (25/248). Regarding the malignant tumors, carcinoma in mixed tumor was the most common histological subtype (77/223), followed by complex carcinoma (49/223), tubulopapillary carcinoma (24/223), tubular carcinoma (21/223), solid carcinoma (16/223), comedocarcinoma (10/223), inflammatory carcinoma (9/223), malignant myoepithelioma (5/223), micropapillary invasive carcinoma (4/223), carcinosarcoma (3/223), adenosquamous carcinoma (2/223), anaplastic carcinoma (2/223), and mucinous carcinoma (1/223). Regarding the benign tumors, benign mixed tumor was the most common tumor subtype (14/25), followed by simple adenoma (6/25) and complex adenoma (5/25). Patients with inflammatory carcinoma or carcinosarcoma experienced shorter survival times than other patients (*P* < 0.0001). The complete clinical information can be found in Table 1.

**Table 1:** Clinical parameters of the 248 dogs used in this study.

**Variables**	**Benign tumors**	**Malignant tumors**	***P*-value**
Number of cases	25 (10%)	223 (90%)	
Age	9.2 ± 1.99	10.1 ± 2.01	*P* > 0.05
Breed			
Pure	15 (60%)	152 (61.3%)	*P* > 0.05
Mixed	10 (40%)	71 (38.7%)	*P* > 0.05
Ovariohysterectomy			
Yes	0 (0%)	49 (19.7%)	*P* > 0.05
No	25 (100%)	199 (80.2%)	*P* > 0.05
Ulceration			
Absent	25 (100%)	196 (79.1%)	*P* > 0.05
Present	0 (0%)	52 (20.9%)	*P* < 0.05
Histological grade^*^			
I	–	105 (50.9%)	–
II	–	60 (29.1%)	–
III	–	41 (20%)	–

* *Histological grading included only, 206 dogs*.

Forty-two of the 248 canine mammary samples were not histologically graded since they were samples of benign tumor (25) or a special tumor subtype (17). Regarding the 206 graded tumor samples, grade I tumors were the most frequent (105/206), followed by grade II (60/206) and grade III (41/206) tumors. Unsurprisingly, the patients with grade III tumors experienced the shortest survival times (*P* < 0.001), followed by the patients with grade II tumors and the patients with grade I tumors. Regarding the lymph node status, in 14 out of 248 patients, lymph node histopathology was not performed. Thirty-two patients had lymph node metastasis at the time of diagnosis. Patients showing lymph node metastasis at diagnosis experienced shorter survival times than patients without lymph node metastasis (*P* < 0.0001).

### CD31/PAS Double Staining

**Figure 2:**
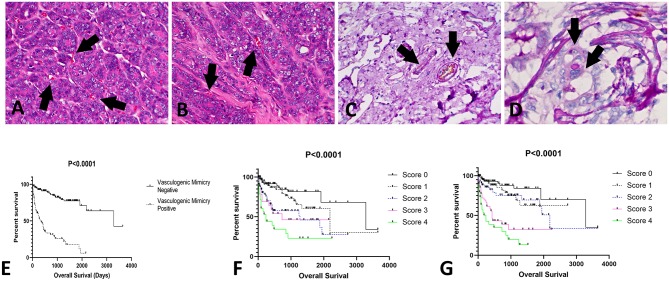
Evaluation of vasculogenic mimicry (VM) in canine mammary gland tumors. **(A,B)** Histological appearance of tubular—like structures formed by neoplastic cells with red blood cells in the lumen (arrows); 200×. **(C)** Positive internal control for CD31/PAS double staining. Note the double positivity for both CD31 (brown staining) and PAS (pink staining) (arrows); 200×. **(D)** Tubular-like structure (arrows) formed by neoplastic epithelial cells positive for PAS; 400×. **(E)** Overall survival of patients with tumors positive or negative for VM structures. Patients with tumors presenting with VM experienced shorter survival times (*P* < 0.0001). In addition, we identified associations of VEGF-A and VEGFR-2 expression with survival time. Patients with higher scores (3 or 4) for VEGF-A **(F)** or VEGFR-2 **(G)** experienced shorter survival times.

Among all CMT samples (*N* = 248), VM was identified in 33% of the tumor samples (82/248). The presence of VM had a stronger relation with tumor grade than with histological subtype (Figure 2). Thus, only tumors with a higher grade (II or III), independent of tumor subtype, presented VM-positive structures. Additionally, the patients that were positive for VM structures experienced shorter survival times than the negative patients (*P* < 0.0001) (Figure 2).

### VEGF-A and VEGFR-2 Immunostaining

**Figure 3:**
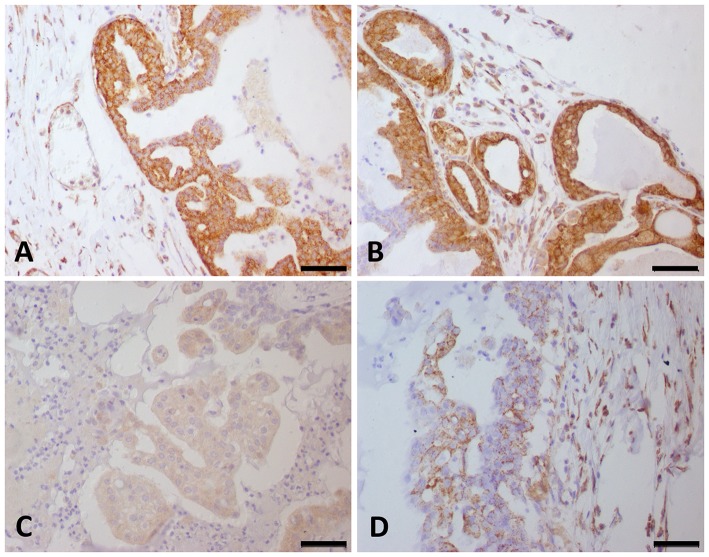
VEGF-A and VEGFR-2 expression in canine mammary gland tumor samples. Strong (score 4) VEGF-A **(A)** and VEGFR-2 **(B)** expression was observed in the same tumor samples. **(C)** Canine mammary gland tumor showing a score of 1 for VEGF-A expression. **(D)** Canine mammary gland tumor showing a score of 2 for VEGFR-2 expression.

Due to the evidence of several pathways involving tyrosine kinase binding, including the *VEGF* pathway, we evaluated VEGF-A and VEGFR-2 expression in a large number of CMT samples. VEGF-A and VEGFR-2 expression was identified in endothelial and neoplastic tumor cells. Among all tumor samples, 178 (74%) out of 248 were positive for VEGF-A. Sixth-five samples had a score of 1 (73/178), 43 scored a 2 (52/178), 41 scored a 3 (25/178), and 34 scored a 4 (28/178). Interestingly, the patients with relatively high VEGF-A scores experienced reduced survival (*P* < 0.0001). The VEGF-A score showed a positive correlation with VM. Regarding VEGFR-2 expression, 65 (26%) out of the 248 CMT samples were negative. Among the positive VEGFR-2 samples (*N* = 183), 65 out of 183 had a score of 1, 43 scored a 2, 41 scored a 3, and 34 scored a 4. Patients with a VEGFR-2 score of 4 had the shortest survival times (*P* < 0.0001) (Figure 2). In addition, the samples with a VEGFR-2 score of 3 or 4 were also positive for VM. VEGF-A and VEGFR-2 immunohistochemical staining results are shown in Figure 3.

### Sorafenib IC_50_ and VM *in vitro*

**Figure 4:**
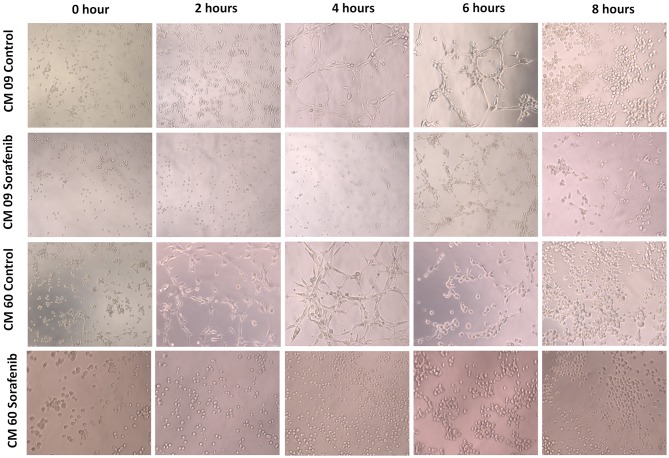
Evaluation of *in vitro* vasculogenic mimicry by two canine mammary gland tumor cell lines (CM9 and CM60). It was possible to observe tubular-like structures in both cell lines after 4 h. After 6 h, both cell lines started to show tubule disruption, and a group of cells had formed at 8 h. The cells treated with sorafenib showed no tubular-like structure formation at 4 h. Additionally, at 6 h, the sorafenib-treated cells had not formed linked tubular structures.

Since we identified *VEGF-A* and *VEGFR-2* downregulation in CMT tumor samples, we investigated the antitumor effect of sorafenib on our CMT cells. Sorafenib has been shown to affect the viability of primary cell cultures of the CM9 cell line, showing an IC_50_ of 2.61 μM, and sorafenib has an IC_50_ of 1.34 μM for CM60 cells. Both cell lines also showed a VM ability *in vitro* after 4 h (Figure 4). The sorafenib IC_50_ for each cell line was able to inhibit *in vitro* VM, and the treated CM9 and CM60 cell lines lacked the ability to form vascular-like structures *in vitro* (Figure 4).

## Discussion

This paper describes the correlations of VM with prognostic factors in female dogs harboring mammary gland tumors. Interestingly, the dogs with tumors that were positive for VM formation exhibited reduced survival times, indicating that VM is an independent prognostic factor in CMT. This feature can be evaluated in HE slides, bringing a new histological tool for determining patient prognosis in CMT. Previously, VM was demonstrated in female dogs with mammary gland tumors (3, 7). However, these previous studies investigated VM only in inflammatory mammary carcinomas (3, 7). Overall, VM was associated with a high tumor grade and an undifferentiated histological subtype. In other types of tumors, such as human hepatocellular carcinomas, VM has been associated with an advanced tumor grade, invasion, metastasis and a short survival time, indicating VM occurs in relatively aggressive tumors (23). In human breast cancer (BC), VM was previously associated with poor patient outcomes and trastuzumab resistance in HER-2-positive tumors (24). Thus, new studies evaluating VM might provide a new therapeutic perspective. Since dogs are considered a model for human BC studies (2), dogs and humans can benefit from comparative oncology initiatives.

We identified by immunohistochemistry a strong correlation between VEGF-A and VEGFR-2 in our tumor samples. In addition, our linear regression analysis demonstrated a dependency between VEGF-A and VEGFR-2 expression. Thus, these findings are evidence that VEGFR-2 expression is dependent on VEGF-A expression by neoplastic cells. VEGFR-2 deregulation induces VM by autophagy (25), cancer stem cell activation (17) and hypoxia (11). Our results indicated that both VEGF-A and VEGFR-2 had associations with VM formation and patient overall survival. Since VEGFR-2 activation leads to the induction of vascular formation (17), VEGFR-2 expression occurs in normal endothelial cells during physiological vasculogenesis. However, cancer cells can also express VEGFR-2 to promote intratumoural vessel formation. In human glioma patients, VEGFR-2 was implicated as a key protein for VM and associated with a poor prognosis. In dogs, VEGFR expression has been investigated in CMT (2, 26, 27). However, no previous studies associated VM with VEGFR-2 expression. As previously demonstrated in human gliomas (17), we believed that the VEGF-A/VEGFR-2 autocrine feed-forward loop could be involved in VM formation in CMT. Thus, we investigated the ability of a VEGFR-2 tyrosine kinase inhibitor (sorafenib) to prevent VM *in vitro*.

To investigate cell viability after sorafenib treatment, we determined IC_50_ values using an MTT assay. Interestingly, the IC_50_ values for our CMT cell lines were lower than those previously reported in the literature for different human cancer cells (28–31). This result reinforces the use of sorafenib in dogs with CMT as a preclinical model for human BC and as a therapeutic option for dogs with relatively aggressive CMT. Sorafenib toxicity and pharmacokinetics were previously investigated in dogs, demonstrating that sorafenib is safe in dogs with cancer (14). Our cancer cell VM structures were evaluated after a 4-h assay, and sorafenib inhibited structure formation. Thus, future clinical trials in dogs can elucidate whether sorafenib is effective in dogs with tumors showing VM.

Several clinical trials have been performed to evaluate sorafenib efficiency in prolonging patient survival; however, the results are controversial (32–36). Overall, the combination of sorafenib with chemotherapy or endocrine therapy has produced clinical improvements in patients. One important limitation of these previous studies is the inclusion criteria limiting the study population to only patients with advanced disease, with no predictive marker selecting which patients will benefit from the therapy (37). In this scenario, our study proposes that breast cancer-affected patients with histological evidence of VM can benefit from sorafenib treatment. However, prior to using VM as a marker favoring sorafenib treatment, a clinical study in CMT-affected dogs is necessary to provide stronger evidence for sorafenib use in clinical practice.

Since dogs with spontaneous canine mammary gland tumor can be an important model of human breast cancer, it is important to perform clinical studies in owned dogs, but it would not be ethical to use sorafenib in these dogs without prior evidence that VM can be inhibited by sorafenib. Thus, our study is the first preclinical study to show evidence that sorafenib can target cells with a VM ability.

## Conclusion

Our results strongly suggest that VM is a prognostic factor in female dogs with mammary gland tumors and is related to a shortened survival time. VM formation can be induced by *VEGFR* deregulation, opening a new perspective for treatment with specific inhibitors. We found that sorafenib inhibited VM *in vitro* and had an antitumoral effect, supporting its use in future clinical trials involving dogs.

## Data Availability Statement

All datasets generated for this study are included in the article/supplementary material.

## Ethics Statement

The animal study was reviewed and approved by Institutional Ethics Committee for the Use of Animals (protocol number: CEUA 0208/2016). Written informed consent was obtained from the owners for the participation of their animals in this study.

## Author Contributions

MP, PF, AL-F, and CF-A conducted all *in vitro* experiments. SM, GG, and CF-A retrieved the paraffin blocks from the pathology service, performed immunohistochemistry experiments, and conducted the survival analysis. MP, RL-A, and CF-A contributed to the experimental design, intellectual input, and data interpretation. MP and CF-A wrote the manuscript. All authors revised the final version of this manuscript.

### Conflict of Interest

The authors declare that the research was conducted in the absence of any commercial or financial relationships that could be construed as a potential conflict of interest.
